# Segregating *YKU80* and *TLC1* Alleles Underlying Natural Variation in Telomere Properties in Wild Yeast

**DOI:** 10.1371/journal.pgen.1000659

**Published:** 2009-09-18

**Authors:** Gianni Liti, Svasti Haricharan, Francisco A. Cubillos, Anna L. Tierney, Sarah Sharp, Alison A. Bertuch, Leopold Parts, Elizabeth Bailes, Edward J. Louis

**Affiliations:** 1 Institute of Genetics, Queen's Medical Centre, University of Nottingham, Nottingham, United Kingdom; 2 Baylor College of Medicine, Houston, Texas, United States of America; 3 The Wellcome Trust Sanger Institute, Hinxton, United Kingdom; Princeton University, United States of America

## Abstract

In yeast, as in humans, telomere length varies among individuals and is controlled by multiple loci. In a quest to define the extent of variation in telomere length, we screened 112 wild-type *Saccharomyces sensu stricto* isolates. We found extensive telomere length variation in *S. paradoxus* isolates. This phenotype correlated with their geographic origin: European strains were observed to have extremely short telomeres (<150 bp), whereas American isolates had telomeres approximately three times as long (>400 bp). Insertions of a *URA3* gene near telomeres allowed accurate analysis of individual telomere lengths and telomere position effect (TPE). Crossing the American and European strains resulted in F1 spores with a continuum of telomere lengths consistent with what would be predicted if many quantitative trait loci (QTLs) were involved in length maintenance. Variation in TPE is similarly quantitative but only weakly correlated with telomere length. Genotyping F1 segregants indicated several QTLs associated with telomere length and silencing variation. These QTLs include likely candidate genes but also map to regions where there are no known genes involved in telomeric properties. We detected transgressive segregation for both phenotypes. We validated by reciprocal hemizygosity that *YKU80* and *TLC1* are telomere-length QTLs in the two *S. paradoxus* subpopulations. Furthermore, we propose that sequence divergence within the Ku heterodimer generates negative epistasis within one of the allelic combinations (American-*YKU70* and European-*YKU80*) resulting in very short telomeres.

## Author Summary

Telomere length is a complex trait that varies among individuals. Its regulation is critical to the process of aging, and altered length control can result in either senescence or immortalization. We detected extreme variation between different subpopulations of the wild yeast *S. paradoxus*, the closest relative to *S. cerevisiae*. By tagging individual telomeric ends in these two groups, we show that regardless of the total number of telomeric repeats, the critical length at which any telomere is replenished remains conserved. To detect the quantitative trait loci (QTLs) behind the length variation, we used the two sub-populations with the most polar distribution to generate progeny and perform linkage analysis. Further, we validated that naturally occurring sequence variations in *YKU80* and *TLC1*, two genes previously shown to be important for telomere length maintenance, can explain part of the variation. We also identified other loci that influence both telomere length and gene silencing. Further investigation will provide more insights into the underlying genetic mechanism behind normal telomere regulation, potentially relevant in aging and aging-related disease such as cancer.

## Introduction

Telomeres are active nucleo-protein sites that constitute the ends of chromosomes in most eukaryotic species [1]. Telomeric DNA is G-rich and highly species specific in sequence. The telomeric DNA is bound by a number of proteins that together form a tight structure that effectively hides chromosome ends from DNA repair enzymes. Telomeric sequences are elegantly replenished by telomerase, a specialized reverse transcriptase. This process compensates for the loss of DNA from chromosome ends during DNA replication. Telomere length is both species and chromosome specific and appears to play an important role in the normal functioning of a cell. In *S. cerevisiae*, the mean telomeric length is ∼350 bp, whilst in humans it is several kilobases [1]. Although telomere length is generally conserved and maintained under quite rigorous control, different isolates of *S. cerevisiae* display moderate variation [2],[3]. Similar evidence of variation has been documented between individuals of worm [4], mouse [5], plant [6] and human [1] species. In *S. cerevisiae*, analysis of a complete set of deletion mutants revealed over 150 genes that altered telomere length [2],[7]. Telomerase (including its RNA template *TLC1*), Rap1, yKu, and Mre11 complexes, Pif1p and Cdc13p are amongst the principal regulators of telomere length.

In this study, we investigated variation in telomere length among 112 *Saccharomyces* isolates, mostly belonging to the *S. cerevisiae* and *S. paradoxus* species, previously characterised for their subtelomeric structure and Ty prevalence [8]. Although the two species share many features, their histories differ notably. *S. cerevisiae* has been used for thousands of years by humans. Selection and domestication issues have been previously invoked [9],[10] to argue that *S. cerevisiae* may not be a good model for studying natural variation of complex traits. In contrast, *S. paradoxus* has had no such interaction with humans. Sequence analysis in this species supports the presence of three major geographic subpopulations: European, Far Eastern and American [8], [11]–[14]. Subtelomeric structures are also highly variable between *S. paradoxus* subpopulations as illustrated by the documented paucity of the Y′ element in Far Eastern isolates [8]. The overall sequence variation found in *S. paradoxus* (up to 4.5%) is much greater than in any *S. cerevisiae* analysed (∼0.7%) [14]. The results reported below describe the phenotypic telomeric differences among *Saccharomyces* strains and the genetic mechanisms underlying this variation.

## Results

### Extreme Telomere Length Variation in *S. paradoxus* Strains

**Figure 1:**
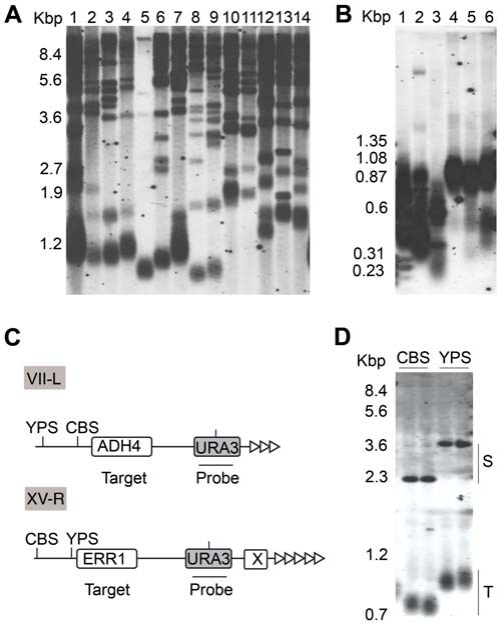
Telomere length screening in *Saccharomyces* strains. (A) *Xho*I digestion of genomic DNA of several *S. cerevisiae* and *S. paradoxus* strains probed with telomeric TG_1–3_ repeats. *S. cerevisiae*: 1) S288c, 2) DBVPG6763, 3) Y55, 4) SK1, 5) DBVPG6693, 6) YPS128, 7) DBVPG1108; *S. paradoxus*: European, 8) CBS432, 9) N-17; Far Eastern, 10) NBRC1804, 11) N-44; North American, 12), YPS125, 13) YPS138, 14) DBVPG6304. (B) Genomic digestion using enzymes that do not digest within telomeric repeats. *S. cerevisiae*: 1) S288c, 2) Y55; *S. paradoxus*: 3) CBS432, 4) N-44, 5) N-45, 6) N-46. (C) Single telomere tag of chromosomes VII-L and XV-R. Insertion point of the *URA3* is indicated (target). Probes (horizontal line) and *Sca*I restriction sites (vertical line) are indicated. (D) Southern blot of genomic DNA digested with *Sca*I and probed with *URA3*. Two independent transformants of CBS and YPS tagged at the VII-L telomere and propagated for 250 generations are shown. S indicates the internal fragment and T the fuzzy telomere end. Subtelomeric polymorphic restriction sites generate different restriction fragments in the YPS and CBS strains.

Two different genomic DNA digests hybridised with a TG_1–3_ probe were used to screen telomere length in 112 *Saccharomyces* strains. Representative Southern blots are shown in Figure 1. One digest used *Xho*I, which cuts within the subtelomeric Y′ element approximately 950 bp from the junction of subtelomeric DNA and the terminal telomeric repeats [15]. This element is present at 2/3 of the telomeres in the sequenced strain S288c and is highly variable in number among different isolates [8]. In *S. cerevisiae*, the terminal restriction fragments (TRFs) of Y′-ends are approximately 1.2 Kbp in size (Figure 1A). We also used a mixture of 5 different 4 bp recognition restriction enzymes (Figure 1B), which digest the DNA into very small fragments (16 bp average) but leave the telomeric repeats intact [16]. As previously reported [2],[3], different strains display variation in the length of the telomeric tract (Figure 1). In our study, the general variability of telomere length appeared greater in *S. paradoxus* than in *S. cerevisiae*. For example, three widely used *S. cerevisiae* lab strains, S288c, Y55, and SK1 (Figure 1A), showed little variation of telomere length at Y′-ends although a brewing strain, DBVPG6693, had telomeres significantly shorter than the other *S. cerevisiae* strains. In contrast, *S. paradoxus* strains varied greatly in their TRFs (Figure 1A and 1B). This variation correlated with their geographic origin. European strains of *S. paradoxus* appeared to have short telomere tracts when compared to the Far Eastern and American isolates. Moderate variation was seen within the geographic clusters.

### Telomere Length Is Background-Dependent

The analysis of TRFs by Southern blot allows the screening of a large number of samples but also manifests some drawbacks. Variation in the Y′ element number or sequence makes this method liable to generate artefacts which can complicate the interpretation of results. In order to validate our initial screening, we precisely measured telomere length at individual telomeres by inserting an identical and unique sequence adjacent to the telomeric repeat. Three strains representative of the major *S. paradoxus* subpopulations, CBS432 (European), YPS138 (American), referred from here as CBS and YPS respectively, and N44 (Far Eastern), were tagged at telomeres VII-L and XV-R using a *URA3* marker previously deleted in chromosome V (Table S1). At telomere XV-R we targeted the *ERR1* gene, known to be a single copy in *S. paradoxus* [8], using *URA3* marker flanked by 60 bp of *ERR1*, an X-element and 211 bp of TG_1–3_ repeats (Figure 1C). The VII-L end was tagged using the *URA3* marker flanked by *ADH4* and 81 bp of telomeric repeats (Figure 1C). Cells were propagated for 250 generations before telomere length was measured to ensure that the telomere tract at the *URA3* marked telomere had been reset. Consistent with the initial screening, the single VII-L telomere revealed a significant difference in length between the strains: 201±15 bp in CBS and 438±29 in YPS (Figure 1D). The Far Eastern isolate, N44, had a telomere length in-between CBS and YPS (not shown) and was not analysed further. We observed no variation in telomere length among independent transformants of the same strain, different colonies from the same transformant or spores derived from the diploid strain (data not shown). Similarly, telomere length at XV-R showed the expected length difference between the two isolates: 189±53 bp in CBS and 416±43 bp in YPS. These results indicate that the telomere length variation in *S. paradoxus* was reproducible and strain background dependent. Altogether, the genetic background explains 98% of the variability in the phenotype, making it a good candidate for a QTL study considered below.

**Figure 2:**
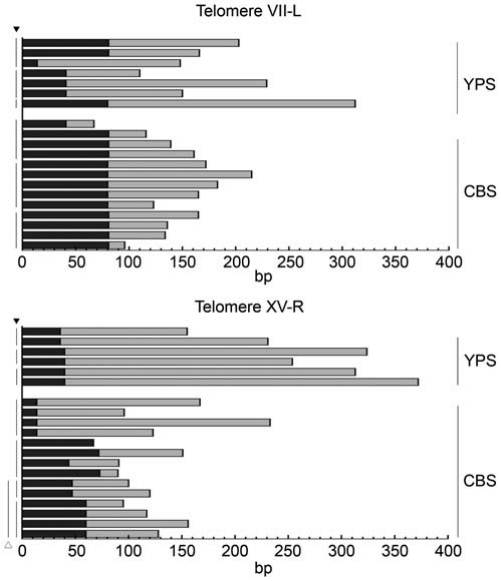
Telomeres length analysis at nucleotide resolution. Individual telomere were cloned and sequenced. Conserved telomere core (black) and *de novo* telomerase added (grey) repeats are shown for VII-L and XV-R in several CBS and YPS clones. The black arrow indicates clones from the same DNA preparation in independent transformants. The white arrow indicates clones from a single transformant independently propagated for 200 generations.

The *URA3* marked telomeres were further used to analyse telomere length at the nucleotide level using a C-tail mediated cloning and sequencing approach [17]. We sequenced several clones from three independent transformants as well as from colonies independently propagated from single transformants for both CBS and YPS (Figure 2). As previously shown, DNA cloned from each preparation, which originated from a population of cells, exhibited divergence in shortening as well as of telomerase extension [18]. Although sequencing across telomeric repeats proved difficult, clones derived from the CBS background, having the shortest telomere tracts, were fully sequenced in most of the cases (25/27). Telomere length in these clones ranged from 67 to 215 bp (average 148 bp, 11 clones) at VII-L and 67 to 233 bp (average 124 bp, 14 clones) at XV-R.

Given that yeast telomerase adds degenerate repeats to the telomere, the point of telomerase action could be determined by sequence comparison in both strain backgrounds (Figure 2). This point indicates the extent of telomere attrition prior to becoming a substrate for telomerase at single nucleotide resolution. In CBS, the conserved lengths at XV-R varied from 14 bp to 73 bp. In contrast, all but one clone of the CBS VII-L maintained 81 bp of internal TG_1–3_ repeats. Similar results were obtained in the YPS strain (Figure 2). Comparing the length of the conserved core and telomerase-added telomeric repeats gives further insight into the mechanism causing telomere length variation between CBS and YPS strains. The fact that we found no significant differences between the average length of the conserved sequence between CBS and YPS indicates that cells sense critically short telomeres by a conserved mechanism that recognises a specific telomere length. This might indicate that telomere replenishment is a mechanism under evolutionary constraint. It is worth noting that the minimal telomere length detected, inferred from the measure of maximum internal resection (Figure 2, length of black bars), was as short as 14 bp.

### Short Telomeres Are Insensitive to *YKU* Deletion

**Figure 3:**
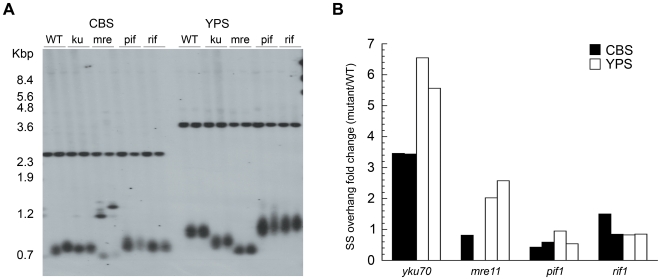
Telomere length and end protection assay in *S. paradoxus* mutants. (A) Effect of *YKU70*, *MRE11*, *PIF1*, and *RIF1* deletion in telomere length at VII-L. Two independent transformants were propagated for 150 generation and genomic DNA was digested and probed with *URA3*. Similar results were obtained at telomere XV-R (not shown). (B) Amount of SS DNA was analysed in the same mutants used in (A) using a telomere G-overhang assay (see Materials and Methods). The results were normalised for the WT and show the fold changes of SS overhang. Black and white column respectively indicate CBS and YPS background.

To gain further insight into telomere length regulation, we deleted four genes known to affect telomere length in both the CBS and YPS strains. *MRE11* and *YKU70* deletions normally result in severe telomere shortening whereas *RIF1* and *PIF1* mutants have extra long telomeres [2],[7]. Spores from the heterozygous knockouts were propagated for 250 generations before telomere length was assessed at VII-L and XV-R ends using a *URA3* probe. Growth defects were observed in both *yku70Δ* and *mre11Δ* in the CBS but not in YPS strain background, indicating that the CBS strain could only partially tolerate mutations that result in a very short telomere phenotype. The telomere shortening was more severe in *mre11Δ* compared to the *yku70Δ* in both strain backgrounds (Figure 3A) whereas in *S. cerevisiae* they are known to have comparable effects [19]. Interestingly, deletion of *yku70* in CBS did not seem to alter telomere length (Figure 3A). Similar results have been obtained from *yku80Δ* (results not shown). In contrast to *yku70Δ*, *mre11Δ* resulted in the dramatic loss of telomeric repeats in CBS with extra bands appearing at 1.2–1.3 kbp on the Southern blot (Figure 3A). These extra TRFs could be a result of recombination events, as the reduction in length could trigger recombination in a similar manner to that seen in certain *Kluveromyces lactis* telomerase mutants with a short telomere phenotype [20]. Deletion of *PIF1* and *RIF1* resulted in the expected telomere elongation and increased heterogeneity in both CBS and YPS.

The insensitivity of telomere length to *yku70* or *yku80* deletion suggested that the Ku heterodimer may be intrinsically defective at end protection in the CBS strain. To test this possibility we measured the amount of single stranded (ss) DNA constituting the telomeric terminal G-overhang in both CBS and YPS (Figure 3B). As expected, the loss of Ku function in the YPS strains resulted in an almost 7-fold increase in G-overhang. Interestingly, we observed a more than 3-fold increase in G-overhang in the CBS-*yku70Δ* mutants suggesting that the Ku heterodimer does, in fact, protect the chromosome ends in the CBS strain. The loss of end protection may also explain the growth defect phenotype observed in the CBS-*ykuΔ* strain. A temperature growth assay also showed a phenotypic effect of *ykuΔ* (Figure S1). These phenotypes clearly indicate that the Ku heterodimer is not fully defective in CBS strain and some functions are evolutionary conserved. A similar pattern of increased level of G-overhang was observed at the telomeres in the YPS *mre11Δ* strains. No differences were detected in the CBS *mre11Δ*, because the severe loss of telomere length made further analysis of G-overhangs not possible. The *rif1Δ* and *pif1Δ* mutants did not show any significant effect on the G-tails of either strain as expected.

### Analysis of F1 Segregants Reveals Several QTLs Are Involved in Natural Telomere Length Variation

**Figure 4:**
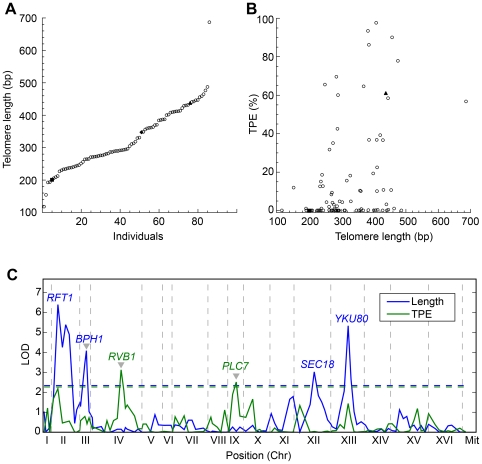
F1 spores phenotypes and linkage analysis. (A) Telomere length distribution in 83 F1 spores (open circles), YPS (solid triangle), CBS (solid square), and the F1 hybrid (solid diamond). A continuum of lengths, consistent with many QTLs, is observed. (B) TPE and telomere length obtained from 83 F1 spores from CBS and YPS cross (open circles). Our results show that the two phenotypes weakly correlate (r = 0.39, linear equation). The two parental YPS (solid triangle) and CBS (solid square) are also shown. (C) LOD plot from linkage analysis for telomere length (blue) and TPE (green) using the normal model and the non-paramentric model respectively. Dashed lines indicate the 5% significance cutoff using 1,000 permutations. Markers with highest LOD in each interval are indicated. Gray arrow indicates QTLs having antagonistic effect.

The large difference seen in telomere length between CBS and YPS made these strains ideal candidates for segregation analysis. F1 hybrids of YPS and CBS were created with telomere lengths that were intermediate between those of the parents (Figure 4A), with little variation between replica crosses and no effect of the original length of the tagged telomeres (results not shown). We generated 84 F1 spores from 21 tetrads from a CBS/YPS F1 hybrid. It is worth noting that the extremely high sequence divergence (3.71%) between the CBS and YPS strains resulted in partial reproductive isolation with only 35% of the gametes being viable [13]. However, at this level of divergence, we could still recover four genuine haploid gametes originating from a single meiotic event. The 84 spores were genotyped at 113 evenly spaced loci, spread across all 16 chromosomes including regions containing major candidate genes for telomere length and silencing. Genotype analysis ruled out genetic incompatibility [21] as a possible mechanism of reproductive isolation between the European and American *S. paradoxus* genes as there are no combinations of CBS and YPS genes located on different chromosomes that are underrepresented among the segregants. We estimated from the genotype data an average of 33.5 crossing over events (CO) per meiosis (results not shown) with many of the chromosomes segregating without a single CO. This CO count is about one third of the CO per meiosis previously detected in a cross between *S. cerevisiae* isolates [22]–[24]. The very high sequence divergence between the CBS and YPS strains accounts for the CO reduction observed [25],[26].

The 84 hybrid progeny were propagated for 200 generations and phenotyped for telomere length at VII-L (Figure S2) and for an additional telomeric trait, namely telomere silencing (discussed below), which is also quantitative. Telomere length analysis revealed a continuum of values (Figure 4A), with an average in the segregants (325 bp) remarkably close to the length measured in the hybrid (347 bp). We found transgressive segregation in 14 F1 spores that had telomeres longer or shorter than the parental telomeres. Polymorphic restriction sites within the subtelomeric region adjacent to the *URA3* insertion allowed identification of the donor of the tagged telomere (Figure 1D and Figure S2). Segregation of the nearby subtelomeric marker proved that the telomere donor had no effect on telomere length. This indicates that there is no epigenetic effect on telomere length regulation and also that no *cis* elements are involved.

Four intervals in chromosomes II, III, XII and XIII were significantly associated with telomere length by linkage analysis (Figure 4C and Table S2A). The interval on chromosome II spans a large section (∼300 Kb), likely to contain multiple linked QTLs (see below), and includes the RNA template of telomerase, *TLC1*. The interval on chromosome III has an antagonistic effect with the CBS allele contributing to the long telomere phenotype in contrast to the short telomere phenotype of the CBS parent (Table S2A). This region (140–200 kb) does not contain any previously identified deletions severely affecting telomere length [2],[7] and further analysis could reveal a new telomere length regulator. The interval on chromosome XII is only 40 Kb away from another telomerase component *EST1*. The chromosome XIII interval peaks on *YKU80*. Using the normal model, the 5 loci detected (considering there are at least two linked loci on chromosome II) explain 56% of the total telomere length variance. It is worth mentioning that there is no overlap between the intervals found here and the ones previously reported in a linkage analysis between two *S. cerevisiae* strains [2].

We performed a two-locus QTL scan in order to detect novel associations and interactions between candidate loci (Figure S3). The four major QTLs described above exhibit significant additive effects at the 5% cutoff level. This result indicates that these four significant intervals are likely to have independent, additive effects on telomere length. Furthermore, there is a significant additive effect between markers on chromosome II, indicating independent linked QTLs within this large interval. We found no significant novel epistatic interaction effects, even at the more permissive 10% significance cutoff.

### *YKU80* and *TLC1* Are Major QTLs for Telomere Length

**Figure 5:**
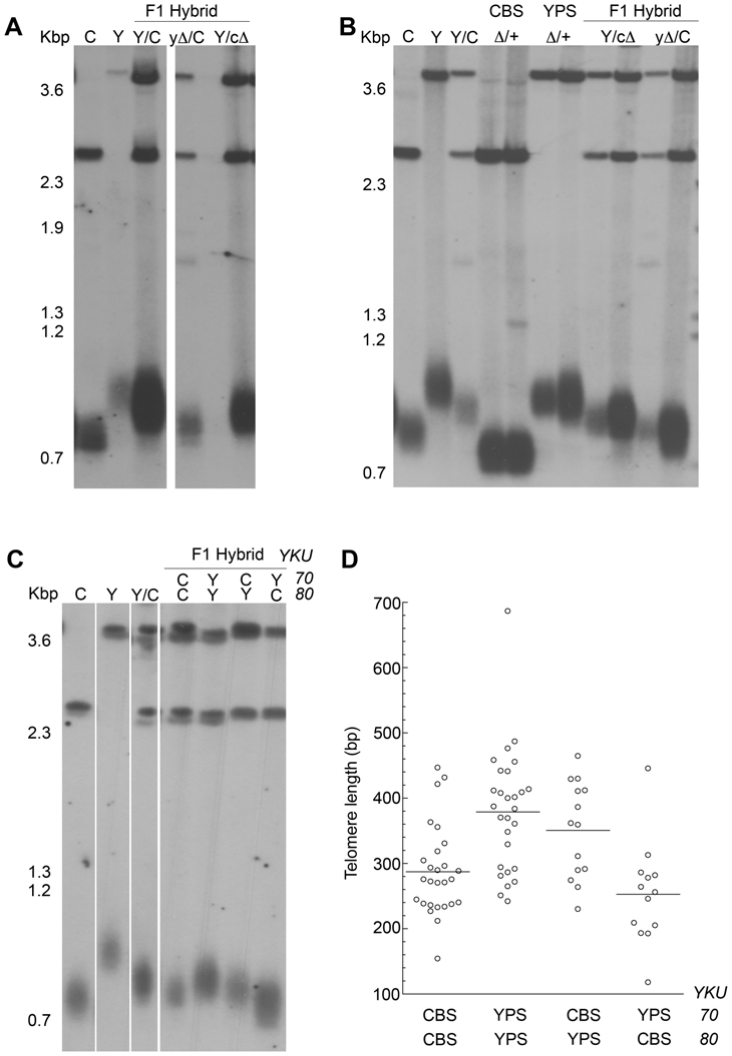
Reciprocal hemizygosity and Epistatic interaction. (A) Telomere length analysis at VII-L using *URA3* as probe. Deletion of the YPS-*YKU80* gene (yΔ/C) result in an F1 hybrid with shorter telomeres compared to when the CBS copy is deleted (Y/cΔ). C (CBS), Y (YPS), Y/C (F1 hybrid). (B) Similar to (A) for *TLC1*. Both parental diploids deleted for one copy of *TLC1* manifest pronounced haploinsufficiency. Reciprocal hemizygosity show shorter telomeres if the YPS-*TLC1* is deleted. Labelling as (A). Δ/+ indicate diploid parental with 1 copy of *TLC1* deleted. (C) F1 hybrids where pairs of *YKU70* and *YKU80* were deleted in all four possible combinations. The combination of YPS-*YKU70* and CBS-*YKU80* shows very short telomeres. (D) Distribution of telomere length for the 83 F1 segregants sorted for the *YKU70* and *YKU80* genotypes. Open circles represent individuals and lines indicate average telomere length of individuals with same *YKU70* and *YKU80* genotypes.

The segregant analysis suggested that the *YKU80* marker was strongly linked with the telomere length phenotype (LOD 5.34, variance explained 12%) with length averages of 370 bp and 276 bp for the spores carrying the YPS or the CBS allele respectively (Table S2). To validate the linkage analysis, we constructed a CBS and YPS F1 hybrid with hemizygous deletions of *YKU80*. We found a telomere shortening effect (78 bp) in the hybrid when the YPS allele of *YKU80* was deleted compared to the deletion of the CBS allele (Figure 5A). Deletion of one copy of *YKU80* in the diploid parental strains showed no telomere length variation, ruling out haploinsufficiency (data not shown).

Similarly, we validated the quantitative effect on telomere length of *TLC1* (LOD 4.25, variance explained 9.74%) by reciprocal hemizygosity (Figure 5B). Both parental diploid strains suffered telomere shortening when one copy of *TLC1* was deleted indicating that the abundance of RNA telomerase template molecules is a limiting factor in telomere length maintenance [27]. The haploinsufficiency effect was more visible in the CBS with 81 bp reduction (42% of total length) than in YPS with 66 bp reduction (14%). The haploinsufficiency effect was also visible in the CBS/YPS hybrid when the CBS or YPS allele was deleted. Deletion of the YPS allele resulted in shorter telomeres when compared to the deletion of the CBS allele with 101 bp (29%) and 38 bp (11%) reduction, respectively (Figure 5B). Two-locus analysis provided no evidence for a significant epistatic interaction between *YKU80* and *TLC1*, however, the two loci showed a significant additive effect (Figure S3). We analysed the RNA sequence and structure of the *TLC1* stem-loop that is known to interact with the Ku heterodimer [28] and found no differences between the two strains (not shown).

### Negative Epistatic Interaction Between CBS-*YKU80* and YPS-*YKU70*

*YKU70* and *YKU80* genes are required for heterodimerization and end binding, and are essential for both telomere protection and NHEJ. The linkage analysis showed strong effect of *YKU80* alleles on the telomere length phenotype but no significant effect of *YKU70*. We experimentally tested if the sequence divergence accumulated in the *YKU* genes in the two strains has an epistatic effect on telomere length. We created F1 hybrids and deleted *YKU70* and *YKU80* in pairs in all four possible combinations (Figure 5C). Interestingly, we detected a detrimental interaction between the CBS-*YKU80* and YPS-*YKU70* in the F1 hybrid resulting in very short telomeres (Figure 5C) comparable in length to telomeres in y*kuΔ* (*yku70Δ/yku70Δ* or *yku80Δ/yku80Δ*) hybrid strains. The deletion of just CBS-*YKU70* in the hybrid did not alter telomere length (data not shown). Analysis of telomere length distribution in the F1 segregants sorted for the *YKU* genotypes did show very short telomere lengths within the same allelic combination, CBS-*YKU70* and YPS-*YKU80* (Figure 5D).

Loss of heterodimerization is one of the possible mechanisms for the negative epistasis of the CBS-*YKU80* and YPS-*YKU70* alleles. Additional possibilities include loss of DNA end binding and alteration of a functional surface generated by both Yku70 and Yku80 that is required for Ku's telomere length regulation function. We mapped the residues that varied between the CBS and YPS *YKU70* and *YKU80* alleles onto the structure of human Ku bound to DNA [29] (Figure S4). Our structural analysis suggests that a few residues may contribute to negative epistasis through either heterodimerization or DNA end binding (Text S1).

We investigated the hypothesis that selection might have played a role in generating this negative epistasis between the CBS-*YKU80* and YPS-*YKU70* by using a population genomic approach [30]. We previously generated a large sequence dataset for *YKU70* and *YKU80* from 46 *Saccharomyces sensu stricto* strains [13]. We estimated *dN/dS* on each branch of the phylogenetic tree [13] for the whole gene and for individual domains (N-terminal alpha/beta domain, central DNA-binding beta-barrel domain and C-terminal arm). The only evidence of positive selection was in the N-terminal domain of *YKU80* on the branch to the European and American *S. paradoxus* isolates. However, we found that the increased *dN/dS* ratio was due to a small number of synonymous changes on this branch in the N-terminal domain and we consider that the most likely explanation is an ancestral polymorphism in *YKU80* in *S. paradoxus*.

Sequence analysis shows that the identity over the whole of chromosome XIII between the European and American *S. paradoxus* strains and between these and the *S. cerevisiae* reference strain S288c is very similar to the corresponding nucleotide identity in *YKU70* (Table 1). *YKU80* on the other hand is more divergent within the *S. paradoxus* species and less divergent between the species. Comparing amino acid identities, *YKU80* is more divergent than *YKU70* within the *S. paradoxus* species and less divergent between species, particularly when the *S. cerevisiae* reference strain is compared to the European *S. paradoxus* strain CBS342. This is confirmed by oriented SNP analysis (Figure S5 and Figure S6), which shows a large number of substitutions on the branch to the ancestor of the American *S. paradoxus* strains in *YKU80* compared to *YKU70*.

**Table 1:** Percentage sequence identity of *YKU* genes.

	CBS *vs.* YPS	S288c *vs.* CBS	S288c *vs.* YPS
Nucleotide			
Chromosome XIII	96.24	87.63	87.19
*YKU70*	96.08	87.73	87.29
*YKU80*	94.31	91.65	90.38
Protein			
Ku70p	96.35	90.22	89.22
Ku80p	93.88	92.91	90.50

Sequence analysis of *S. paradoxus* CBS, YPS, and *S. cerevisiae* S288c strains. Chromosome XIII was used as a control because both *YKU70* and *YKU80* are present in this chromosome in *S. cerevisiae* and *S. paradoxus*.

### Telomere Position Effect Is Quantitative and Only Weakly Correlates with Telomere Length

**Figure 6:**
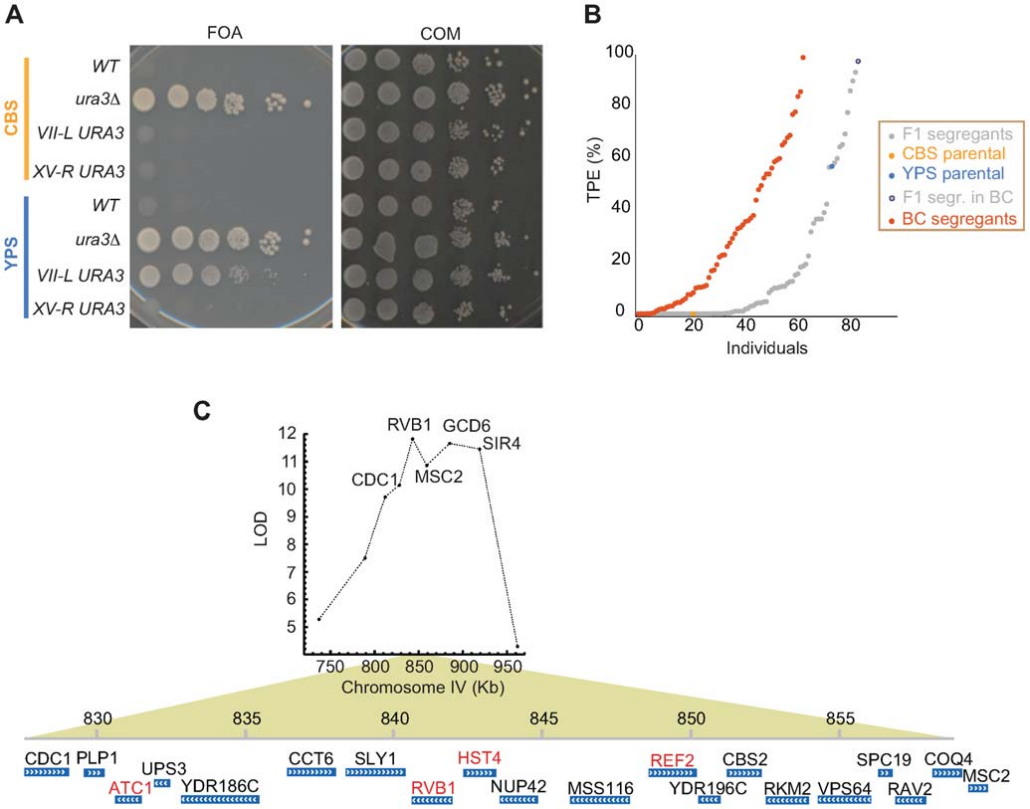
TPE assay, distribution, and mapping. (A) Serial dilutions of cells spotted onto 5-FOA and complete media (COM). No colonies were detected when *URA3* was inserted at CBS telomeres indicating lack of silencing. High silencing was detected at VII-L in the YPS background. Cells were also plated on COM and uracil dropout (not shown) to control for total cell number and *URA3* mutations respectively. (B) TPE results at VII-L telomere. Individuals were ranked for percentage of TPE and each value represents an average of 3 independent replicas. The grey dot series represent segregants from the F1 hybrid obtained by crossing the parental strains CBS (orange) and YPS (blue). The backcross series is represented in red and was obtained by crossing the CBS parental and F1 segregant with highest TPE (grey, black circled). (C) Refined analysis of chromosome IV interval. TPE was measured in a total of 211 segregants genotyped for 9 loci spanning 200 kb on chromosome IV. The LOD value peak at *RVB1* and the 30 kb region around this marker is displayed. Potential candidate genes are indicated in red. A second peak between *GCD6* and *SIR4* markers also indicates a second linked QTL.

Genes close to chromosome ends are subjected to gene silencing or TPE [31]. TPE was assessed by measuring *URA3* expression using the 5-fluoro-orotic acid (5-FOA) plate assay at both XV-R and VII-L (Figure 6A). No TPE was observed in either strain at XV-R indicating that this is one of the non-repressive ends [32]. TPE was observed at the VII-L telomere (61.1±28% of the colonies FOA resistant) in YPS indicating that this mechanism is evolutionarily conserved between *Saccharomyces spp*. The short length of telomeric tract can explain the absence of silencing in the CBS strain, as telomere length negatively regulates the expression of nearby genes by recruiting Rap1p and the SIR complex [31]. In order to test the correlation between TPE and telomere length, we measured TPE in the same set of segregants characterised for telomere length. Our results indicate only a very weak correlation between TPE and telomere length (r = 0.36) with many of the spores exhibiting no TPE (Figure 4B and Figure 6B). Spores with long telomeres and no TPE were found. However, no spores with very short telomeres and a high level of TPE were found indicating that a certain number of telomeric repeats are required to establish silencing. The fact that half of the F1 spores had no TPE could be explained by the presence of one allele in CBS defective for silencing. According to this model we would expect at least 2 spores within each tetrad to lack TPE. However, in 5 of the 21 tetrads three of the four spores exhibited TPE, thus ruling out this simple hypothesis.

Linkage analysis indicates several markers associated with the TPE phenotype (Figure 4C). Because the TPE measurements are strongly non-normal, we used a nonparametric method for assessing the strength of a QTL (Table S2B). TPE phenotype does not exhibit strong genetic associations compared to telomere length. There are two significant QTLs at the 5% significance cutoff on chromosomes IV and IX. The strong QTLs for length on chromosomes II and XIII are also associated with high levels of TPE, but are not significant at the 5% level. We also found several CBS markers (*RVB1*, *SIR4*, *STE4*, *PCL7*) that showed a medium antagonistic effect (Table S2B) in agreement with several F1 segregants with higher TPE than the YPS parental (Figure 6B). To test the presence of several CBS alleles conferring TPE, we backcrossed an F1 spore with the highest TPE (likely to carry all the high TPE alleles from both CBS and YPS) to the CBS parental strain and analysed TPE in 64 backcross progeny (Figure 6B). We observed a significant change in the phenotype distribution with much higher TPE levels in the backcross compared to the F1 progeny likely due to high TPE CBS alleles now present in all progeny. This phenotype distribution also supports the idea of widespread CBS/YPS negative epistasis present in the F1 spores, resulting in no TPE, and now diluted in the backcross.

In order to dissect the QTL intervals in chromosome IV and XIII we refined the genotype analysis within this region. We also generated an additional set of 92 segregants from the CBS-YPS F1 hybrid and a set of 96 segregants from another backcross between the CBS parent and an F1 spore with high TPE (86%) and YPS sequence for both chromosome IV and XIII QTLs. We genotyped the 188 new segregants within the chromosome IV and XIII regions and selected 128 of them, obtained from 32 tetrads that show recombination within the QTL interval, for TPE analysis. We validated that both chromosome IV and XIII intervals contain TPE QTLs using the Wilcoxon-Mann-Whitney rank sum test (*p*<0.0001). Chromosome IV QTL was mapped within a 31 Kb region (between *CDC1* and *MSC2*) with an LOD value peak at *RVB1* (Figure 6C). There are 20 genes within this region with several being possible candidates for the QTL (*HST4*, *ATC1*, *REF2*, *RVB1*). There is also evidence of a second linked QTL that maps within the adjacent *GCD6-SIR4* region.

Similarly, we mapped the QTL interval in chromosome XIII to within a 77 kb region (*YMR087w-STO1*) that peaks at *YKU80*. This gene is likely to be involved in both telomere length and TPE quantitative variation, however TPE phenotype cannot be tested easily using the reciprocal hemizygosity assay because TPE is strongly regulated by ploidy [33].

## Discussion

Telomere length shows quantitative differences controlled by a large number of loci fulfilling the definition of a complex trait. Early reports have shown telomere length to vary among *S. cerevisiae* strains [2],[3]. To obtain a broad picture of the extent of variation of this trait within a natural population and to investigate the mechanisms behind telomere length regulation we screened 112 *Saccharomyces* strains. The initial screening indicated extreme telomere length variation between two geographic subpopulations of *S. paradoxus*. We selected two strains, CBS432 and YPS138 as representative of the European and American subpopulations respectively, and accurately measured telomere length at individual telomeres. We detected a massive 3-fold telomere length variation between the strains: ∼150 bp in CBS432 and ∼450 bp in YPS138.

The high sequence divergence with the large majority of SNPs not shared between subpopulations makes genome-wide association studies impossible. We looked at specific candidates where mutations with telomere length effects were previously characterised. In Est2p, the catalytic subunit of telomerase, substitution within a highly conserved domain (glutamic acid residue 76 to lysine in S288c) results in a telomere length increase of 100 bp [34]. We observed a substitution in exactly the same position in the American *S. paradoxus* lineage (glutamic acid residue to glycine) that could partially explain the observed extra long telomeres (Figure S7).

We further characterised the telomeric repeats in the CBS and YPS strains by sequencing individual telomeres, deleting genes involved in telomere maintenance as well as protection, and measuring the G-overhang in WT and mutant strains. One surprising finding in the CBS strain was that inactivation of the Ku heterodimer did not affect telomere length *per se* but increased the length of G-tail. These results indicate that there are natural variants of the Ku proteins, which affect only some of its functions. Similar separation of function mutants have been obtained in the laboratory [35].

To obtain candidate QTLs, we generated 84 segregants from a CBS/YPS cross and performed linkage analysis. We measured length at individual telomeres to obtain a robust phenotype dataset. We also phenotyped the segregants for an additional quantitative telomeric trait, TPE, that also exhibits extreme variation in the CBS (no TPE) and YPS (high TPE) strains. As predicted by the phenotype distribution, both telomere length and TPE variation seem to be controlled by multiple QTLs (Figure 4). Some of the QTLs, like *YKU80*, are shared between the two phenotypes but many others only affect one of the phenotypes explaining the weak correlation between the two traits. Indeed, several mutations within *RAP1*, *SIR3*, *SIR4*, *YKU70* and *TEL1* have been shown to affect either TPE or telomere length independently [31]. Other potential QTLs involved in the length variation and TPE were also detected (Table S2). It is surprising that many alleles from the CBS strain are associated with high TPE suggesting that other alleles in CBS might mask their effect. Although the CBS-*YKU80* reduces TPE in most of the F1 CBS *RVB1-SIR4* spores, it does not completely eliminate TPE (results not shown).

We confirmed that part of the variation in telomere length among individuals is due to natural sequence variants within *YKU80* and *TLC1*. We also show that high sequence divergence in the Ku heterodimer results in a negative epistatic interaction producing extremely short telomeres in one allele combination. A similar negative interaction has been recently documented for alleles of two mismatch repair genes [36],[37]. We have evidence that negative epistasis is widespread in progeny obtained from the highly diverged parental strains CBS and YPS explaining their low fitness when exposed to a multitude of conditions (Warringer J and Liti G, unpublished results). This widespread epistasis could prevent hybridization between the European and American population of *S. paradoxus* in geographic regions where the two populations coexist but do not interbreed [12]. Widespread genetic incompatibility between highly diverged *C. elegans* strains isolated in England and Hawaii has been previously documented [38].

The presence of negative epistasis between diverging subpopulations can represent a powerful tool in mapping novel gene interactions. Indeed, the two *S. paradoxus* strains used in this study to generate the F1 segregants have a level of sequence divergence almost 10-fold higher than in any other *S. cerevisiae* strains previously used in linkage analysis [22],[39], which is consequently likely to result in strong epistatic interactions. The high sequence divergence also has a dramatic impact on gamete viability resulting from the reduced number of CO. This reduction in CO also decreases the resolution of the linkage analysis, requiring a low number of markers to generate the initial genotypes and to map the recombination breakpoints. We analysed segregants from the same meiotic event to ensure that the gametes are genuine meiotic products, with no aneuploidies, and provide important information on phenotype inheritance. Why geographic subpopulations of the same species have evolved dramatic differences in telomeric properties remains unclear.

## Materials and Methods

### Yeast Strains

Wild type *S. cerevisiae*, *S. paradoxus* and others *sensu stricto* strains used for the telomere length screening were previously described [8]. Strains originated for this work are listed in Table S1. Single telomere tagging of the VII-L and XV-R telomere was obtained by homologous recombination using 60 bp of unique homology within *ADH4* and *ERR1*. The *ADH4* plasmid with *URA3* and telomeric seed was previously described [40]. Telomere cloning and sequence were performed as previously described [18].

### Telomeric Silencing Assays

Telomeric silencing was tested by plating serial dilutions of cell suspensions on complete synethic (COM), 5-FOA and uracil drop-out media and incubated at 30°C as previously described [32]. Haploid, *hoΔ*, versions of the strains were constructed as silencing is reduced in diploid cells [33].

### Southern Blot and G overhang Analysis

Genomic DNA extraction and Southern hybridization for telomere length analysis were performed as previously described [41]. A *URA3* probe was obtained by labelling the pLEM3, a plasmid containing the *S. cerevisiae URA3* gene. Telomere length was analysed using ImageGauge (FUJIFILM). Telomere length values in the text indicate average of at least 3 independent replicates±standard deviation. Yeast genomic DNA was extracted and subjected to G-overahang analysis as previously reported [42]. Fold change was quantified by analyzing intensity of each G-tail and telomeric band. The telomeric non G-tail band is consistently seen at ∼3 Kbp in all CBS and 4 Kbp in all YPS strains. The telomeric band intensity was used as a normalizing factor, thereby serving as a loading control for each lane, as well as a control for the number of telomeric repeats for each genotype. The normalized bands, which provide a ratio of G-tail overhang to total telomeric length, were then analyzed for fold-changes in intensity in comparison to the average of the WT bands in each strain. The G-overhang signals were quantified using Phosphorimager and ImageQuant analysis.

### F1 Spores Genotyping

We previously sequenced the CBS432 and YPS138 genomes to obtain SNPs markers [14], with which we generated a linkage map of 113 loci. We genotyped 83 F1 segregants obtained from 21 four-viable tetrads. One spore died after re-streaking from the dissection plate. The genotyping was obtained by high resolution melting PCR (HRM-PCR) using the Corbet Rotorgene and Quantace PCR HRM mix. Primer sequences are available upon request.

### Phylogenetic Methods

Sequences were aligned using ClustalX [43]. Non-synonymous and synonymous rate ratios and ancestral sequences were inferred by the maximum likelihood method in the program CODEML from the PAML package [44] using tree topologies estimated by the neighbor joining method implemented in Clustal X [43].

### Linkage Analysis and Statistics

Linkage analysis was done using the rQTL software [45]. Statistical comparisons were made using the Student t Test for unpaired data with unequal variance and Wilcoxon-Mann-Whitney test provided in KaleidaGraph (Synergy Software).

## Supporting Information

Figure S1 Temperature growth assay. Serial dilutions of cells plated in YPD and incubated at three different temperatures. The CBS strain is temperature sensitive and does not grow at 37°C. Using a less restrictive temperature, 35°C, deleting either *YKU70* or *YKU80* gives a 10–100 fold effect compared to WT. The temperature sensitivity effect is also clear in the YPS strain background at 37°C.
(2.24 MB TIF)

Figure S2 Telomere length in F1 spores at VII-L. A representative Southern blot for telomere length analysis of CBS (C), YPS (Y), and seven tetrads (T1 to T7) from the CBS×YPS F1 hybrid is shown. The 2∶2 segregation of the subtelomeric marker (except for T3, 3∶1) can be clearly seen.
(6.02 MB TIF)

Figure S3 Epistatic interactions. Heatmap of additive (top left) and interaction effect (bottom right) LOD scores for telomere length. Colour column bar indicates the colour-coded LOD value. The 5% significance cutoffs from permutations are 4.36 and 3.96 for the additive and interaction models, respectively.
(5.70 MB TIF)

Figure S4 Structural distribution of CBS and YPS Yku70 and Yku80 variant residues. Yku70 (blue) and Yku80 (red) variant residues are shown as spheres mapped onto a cartoon structure of human Ku (grey) bound to DNA (black ribbon). The central figure shows the β-barrels, DNA rings and C-terminal arms of the subunits in the absence of the N terminal α/β domains. The variant residues that might impact on DNA binding or heterodimerization are circled on the top and bottom of the structure, respectively.
(2.60 MB EPS)

Figure S5 Inferred protein sequences of *S. cerevisiae* and *S. paradoxus* ancestors of *YKU70* and *YKU80*. Alignment of ancestral sequences inferred from the large data set of *YKU70* and *YKU80* sequences from *Saccharomyces sensu stricto* strains (see main text) excluding *S. mikatae* NBRC 1815. Sites with a gap in any sequences were discarded. Sp_Sc_anc is the inferred sequence of the ancestor of both *S. cerevisiae* and *S. paradoxus* (i.e. the ancestor at the node marked by the black dot in Figure S6), Sc_anc is the inferred sequence of the ancestor of *S. cerevisiae* strains (yellow dot in Figure S6), Sp_anc of *S. paradoxus* strains (blue dot in Figure S6), Sp_Eu_anc is the inferred ancestor of the European *S. paradoxus* strains (green dot in Figure S6) and Sp_Am_anc of the American *S. paradoxus* strains (orange dot in Figure S6). Coloured residues in the alignment indicate that a substitution occurred on the branch to that ancestor. For example, at site 2 in panel A the residue present in the ancestor of *S. paradoxus* strains is glutamine (Q) and the residue at site 2 in the ancestor *S. cerevisiae* and *S. paradoxus* is glutamine, but the residue at site 2 in the ancestor of *S. cerevisiae* strains is arginine (R), so the substitution from glutamine to arginine occurred on the branch to *S. cerevisiae*, i.e. the branch joining black and yellow dots in Figure S6.
(0.52 MB EPS)

Figure S6 Phylogenetic analysis of *YKU70* and *YKU80*. Number of amino acid substitutions on each branch of the tree joining the geographically distinct *S. paradoxus* strains and *S. cerevisiae* strains inferred from Figure S5 for *YKU70* and *YKU80*.
(0.28 MB EPS)

Figure S7 Phylogenetic analysis of Est2. Amino acid (AA) sequences of Est2 region I in *S. cerevisiae*, *S. paradoxus*, and *S. mikatae*. Dots indicate identities to S288c sequence. AA substitution at position 76 increases telomere length in *S. cerevisiae* S288c. A substitution at same position is observed in the North American *S. paradoxus* YPS138, which also present long telomeres. This AA substitution is specific to the American lineages of *S. paradoxus* and originated after the split of the American and European/Far Eastern *S. paradoxus*.
(0.44 MB EPS)

Table S1 Strains used in this study. Information about the genotype of the strains is listed. Additional information on the geographic origin and sources of the wild type isolate has been previously reported [8].
(0.07 MB DOC)

Table S2 Markers with significant linkage to telomere length and TPE phenotypic variation. (A) Markers potentially linked to QTLs involved in CBS and YPS telomere length variation. Markers were designed within ORFs and name, chromosomes and coordinate position are listed. LOD values and variance explained were calculated using the normal model. Mean of telomere length for CBS and YPS alleles is shawn with t probability calculated using the student t-test with unpaired data unequal variance. (B) Similar to panel A for markers linked to TPE. LOD and variance explained are calculated using the non-parametric model. Median for the CBS and YPS alleles are reported with p value (exact method) calculated using the Wilcoxon-Mann-Whitney rank sum test for unpaired data.
(0.05 MB DOC)

Text S1 Structural analysis of the yKu heterodimer.
(0.03 MB DOC)
