# Supplementary figures and images for: Segregating YKU80 and TLC1 Alleles Underlying Natural Variation in Telomere Properties in Wild Yeast
Source: PLoS Genet. 2009 Sep 18;5(9):e1000659. doi: 10.1371/journal.pgen.1000659 (PMC2734985; doi:10.1371/journal.pgen.1000659)

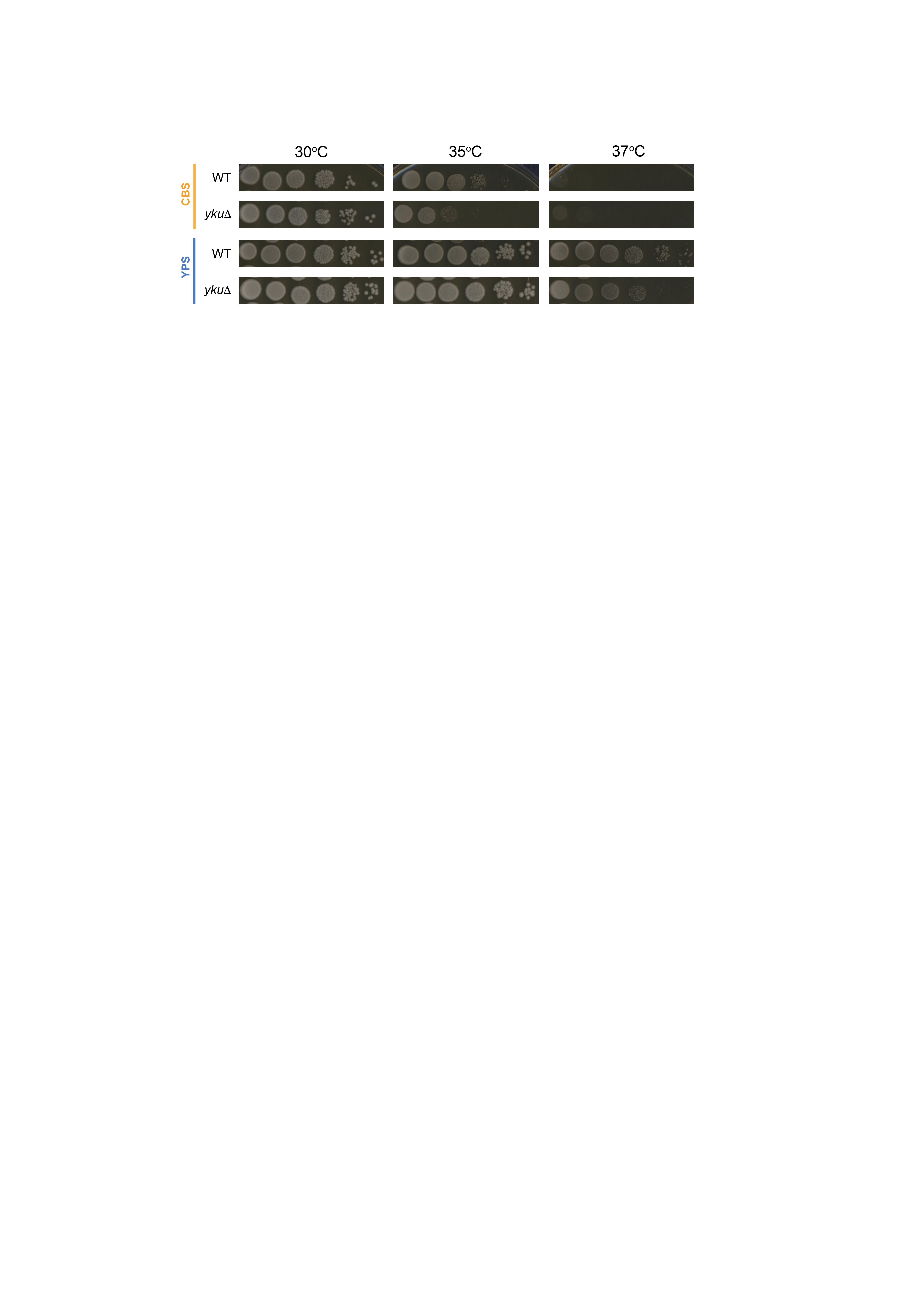

Supplement: Figure S1 — Temperature growth assay. Serial dilutions of cells plated in YPD and incubated at three different temperatures. The CBS strain is temperature sensitive and does not grow at 37°C. Using a less restrictive temperature, 35°C, deleting either YKU70 or YKU80 gives a 10–100 fold effect compared to WT. The temperature sensitivity effect is also clear in the YPS strain background at 37°C. (2.24 MB TIF) [file pgen.1000659.s001.tif]

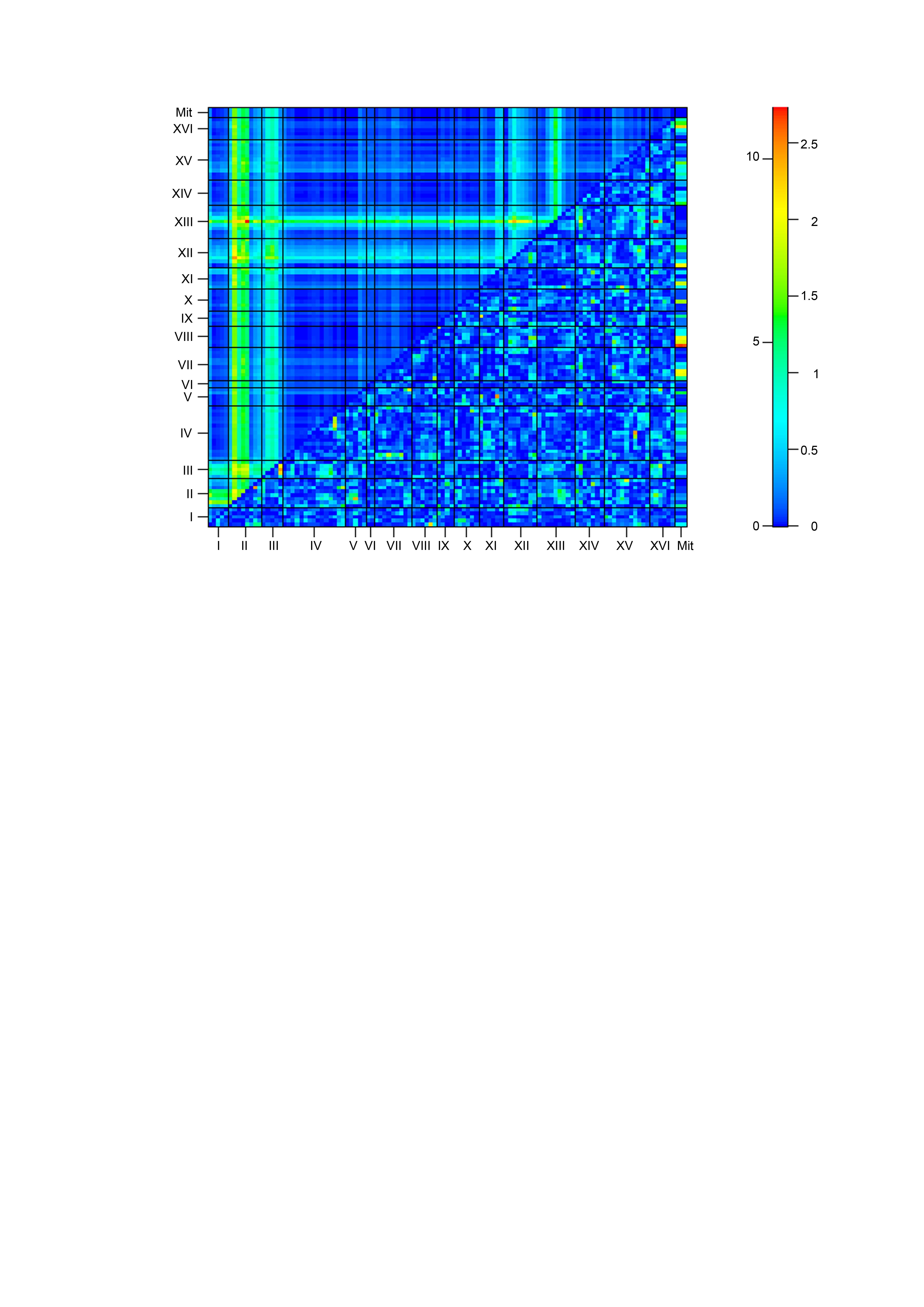

Supplement: Figure S3 — Epistatic interactions. Heatmap of additive (top left) and interaction effect (bottom right) LOD scores for telomere length. Colour column bar indicates the colour-coded LOD value. The 5% significance cutoffs from permutations are 4.36 and 3.96 for the additive and interaction models, respectively. (5.70 MB TIF) [file pgen.1000659.s003.tif]
