# Supplementary material for: Segregating YKU80 and TLC1 Alleles Underlying Natural Variation in Telomere Properties in Wild Yeast
Source: PLoS Genet. 2009 Sep 18;5(9):e1000659. doi: 10.1371/journal.pgen.1000659 (PMC2734985; doi:10.1371/journal.pgen.1000659)
Supplement: Table S1 — Strains used in this study. Information about the genotype of the strains is listed. Additional information on the geographic origin and sources of the wild type isolate has been previously reported [8]. (0.07 MB DOC) [file pgen.1000659.s008.doc]

**Table S1. Strains used in this study.**

| Strain | Genotype and Notes |
| --- | --- |
|  |  |
|  | Strains derived from YPS138 |
| TL192 | *Mat α, ho::Hph, ura3::Kan, URA3 at ADH4 (VII-L)* |
| TL174 | *Mat a/α, ura3::Kan/ura3::Kan, URA3 at ADH4 (VII-L)* |
| TL202 | *Mat a/α, ura3::Kan/ura3::Kan, pif1::Hph/PIF1, URA3 at ADH4 (VII-L)* |
| TL222 | *Mat a/α, ura3::Kan/ura3::Kan, mre11::Hph/MRE11, URA3 at ADH4 (VII-L)* |
| TL238 | *Mat a/α, ura3::Kan/ura3::Kan, rif1::Hph/RIF1, URA3 at ADH4 (VII-L)* |
| TL226 | *Mat a/α, ura3::Kan/ura3::Kan, yku70::Hph/YKU70, URA3 at ADH4 (VII-L)* |
| TL405 | *Mat α, ho::Hph, ura3::Kan, yku70::Nat, URA3 at ADH4 (VII-L)* |
| TL399 | *Mat α, ho::Hph, ura3::Kan, yku80::Nat, URA3 at ADH4 (VII-L)* |
| TL20 | *Mat a/α, ura3::Kan/ura3::Kan, URA3 at ERR1 (XV-R)* |
| TL197 | *Mat a/α, ura3::Kan/ura3::Kan, pif1::Hph/PIF1, URA3 at ERR1 (XV-R)* |
| TL219 | *Mat a/α, ura3::Kan/ura3::Kan, mre11::Hph/MRE11, URA3 at ERR1 (XV-R)* |
| TL218 | *Mat a/α, ura3::Kan/ura3::Kan, rif1::Hph/RIF1, URA3 at ERR1 (XV-R)* |
| TL227 | *Mat a/α, ura3::Kan/ura3::Kan, yku70::Hph/YKU70, URA3 at ERR1 (XV-R)* |
| TL481 | *Mat a/α, ura3::Kan/ura3::Kan, tlc1::NAT/TLC1, URA3 at ADH4 (VII-L)* |
|  |  |
|  | Strains derived from CBS432 |
| TL186 | *Mat a, ho::Kan, ura3::Hph, URA3 at ADH4 (VII-L)* |
| TL154 | *Mat a/α, ura3::Hph/ura3::Hph, URA3 at ADH4 (VII-L)* |
| TL198 | *Mat a/α, ura3::Hph/ura3::Hph, pif1::Kan/PIF1, URA3 at ADH4 (VII-L)* |
| TL205 | *Mat a/α, ura3::Hph/ura3::Hph, mre11::Kan/MRE11, URA3 at ADH4 (VII-L)* |
| TL208 | *Mat a/α, ura3::Hph/ura3::Hph, rif1::Kan/RIF1, URA3 at ADH4 (VII-L)* |
| TL211 | *Mat a/α, ura3::Hph/ura3::Hph, yku70::Kan/YKU70, URA3 at ADH4 (VII-L)* |
| TL408 | *Mat a, ho::Kan, ura3::Hph, yku70::Nat, URA3 at ADH4 (VII-L)* |
| TL402 | *Mat a, ho::Kan, ura3::Hph, yku80::Nat, URA3 at ADH4 (VII-L)* |
| TL154 | *Mat a/α, ura3::Hph/ura3::Hph, URA3 at ERR1 (XV-R)* |
| TL196 | *Mat a/α, ura3::Hph/ura3::Hph, pif1::Kan/PIF1, URA3 at ERR1 (XV-R)* |
| TL203 | *Mat a/α, ura3::Hph/ura3::Hph, mre11::Kan/MRE11, URA3 at ERR1 (XV-R)* |
| TL207 | *Mat a/α, ura3::Hph/ura3::Hph, rif1::Kan/RIF1, URA3 at ERR1 (XV-R)* |
| TL209 | *Mat a/α, ura3::Hph/ura3::Hph, yku70::Kan/YKU70, URA3 at ERR1 (XV-R)* |
| TL486 | *Mat a/α, ura3::Hph/ura3::Hph, tlc1::Kan/TLC1, URA3 at ADH4 (VII-L)* |
|  |  |
|  | Strains derived from crossing CBS432 and YPS138 |
| TL385 | *Mat a/α, ho::Kan/ho::Hph, ura3::Hph/ura3::Kan, URA3 at ADH4 (VII-L)/URA3 at ADH4 (VII-L)* |
| TL426 | *Mat a/α, ho::Kan/ho::Hph, ura3::Hph/ura3::Kan, URA3 at ADH4 (VII-L)/URA3 at ADH4 (VII-L), YPS-yku70::NAT/CBS-YKU70* |
| TL443 | *Mat a/α, ho::Kan/ho::Hph, ura3::Hph/ura3::Kan, URA3 at ADH4 (VII-L)/URA3 at ADH4 (VII-L), CBS-yku70::NAT/YKU70* |
| TL448 | *Mat a/α, ho::Kan/ho::Hph, ura3::Hph/ura3::Kan, URA3 at ADH4 (VII-L)/URA3 at ADH4 (VII-L), YPS-yku80::NAT/CBS-YKU80* |
| TL438 | *Mat a/α, ho::Kan/ho::Hph, ura3::Hph/ura3::Kan, URA3 at ADH4 (VII-L)/URA3 at ADH4 (VII-L), CBS-yku80::NAT/YPS-YKU80* |
| TL465 | *Mat a/α, ho::Kan/ho::Hph, ura3::Hph/ura3::Kan, URA3 at ADH4 (VII-L)/URA3 at ADH4 (VII-L), YPS-yku70::NAT/CBS-YKU70, YPS-yku80::NAT/CBS-YKU80* |
| TL473 | *Mat a/α, ho::Kan/ho::Hph, ura3::Hph/ura3::Kan, URA3 at ADH4 (VII-L)/URA3 at ADH4 (VII-L), CBS-yku70::NAT/YPS-yku70, CBS-yku80::NAT/YPS-YKU80* |
| TL435 | *Mat a/α, ho::Kan/ho::Hph, ura3::Hph/ura3::Kan, URA3 at ADH4 (VII-L)/URA3 at ADH4 (VII-L), YPS-yku70::NAT/CBS-YKU70, CBS-yku80::NAT/YPS-YKU80* |
| TL428 | *Mat a/α, ho::Kan/ho::Hph, ura3::Hph/ura3::Kan, URA3 at ADH4 (VII-L)/URA3 at ADH4 (VII-L), CBS-yku70::NAT/YPS-YKU70, YPS-yku80::NAT/CBS-YKU80* |
| TL483 | *Mat a/α, ho::Kan/ho::Hph, ura3::Hph/ura3::Kan, CBS-tlc1::NAT/YPS-TLC1, URA3 at ADH4 (VII-L)/URA3 at ADH4 (VII-L)* |
| TL485 | *Mat a/α, ho::Kan/ho::Hph, ura3::Hph/ura3::Kan, CBS-TLC1/YPS-tlc1::NAT, URA3 at ADH4 (VII-L)/URA3 at ADH4 (VII-L)* |
