# Supplementary material for: Segregating YKU80 and TLC1 Alleles Underlying Natural Variation in Telomere Properties in Wild Yeast
Source: PLoS Genet. 2009 Sep 18;5(9):e1000659. doi: 10.1371/journal.pgen.1000659 (PMC2734985; doi:10.1371/journal.pgen.1000659)
Supplement: Table S2 — Markers with significant linkage to telomere length and TPE phenotypic variation. (A) Markers potentially linked to QTLs involved in CBS and YPS telomere length variation. Markers were designed within ORFs and name, chromosomes and coordinate position are listed. LOD values and variance explained were calculated using the normal model. Mean of telomere length for CBS and YPS alleles is shawn with t probability calculated using the student t-test with unpaired data unequal variance. (B) Similar to panel A for markers linked to TPE. LOD and variance explained are calculated using the non-parametric model. Median for the CBS and YPS alleles are reported with p value (exact method) calculated using the Wilcoxon-Mann-Whitney rank sum test for unpaired data. (0.05 MB DOC) [file pgen.1000659.s009.doc]

**Table S2A. Telomere length**

| Marker | Chr | Kb | LOD | % Variance explained | Mean  CBS | Mean  YPS | *t* |
| --- | --- | --- | --- | --- | --- | --- | --- |
| *RFT1* | 2 | 183 | 6.40 | 14.29 | 271.1 | 372.1 | <0.0001 |
| *TLC1* | 2 | 308 | 4.25 | 9.74 | 280.8 | 365.4 | <0.0001 |
| *SEC18* | 2 | 399 | 5.39 | 12.18 | 273.6 | 367.7 | <0.0001 |
| *CCZ1* | 2 | 498 | 4.90 | 11.14 | 273.8 | 367.0 | <0.0001 |
| *POL4* | 3 | 140 | 3.46 | 7.99 | 361.8 | 284.7 | <0.0001 |
| *BPH1* | 3 | 183 | 4.09 | 9.39 | 362.8 | 279.4 | <0.0001 |
| *MAT* | 3 | 200 | 3.22 | 7.47 | 360.6 | 285.9 | 0.00014 |
| *SEC13* | 12 | 560 | 3.03 | 7.03 | 287.0 | 359.6 | 0.000222 |
| *Ku80* | 13 | 479 | 5.34 | 12.07 | 275.9 | 369.6 | <0.0001 |
| *TIF34* | 13 | 558 | 2.67 | 6.23 | 288.3 | 356.8 | 0.000442 |

**Table S2B. Telomere position effect**

| Marker | Chr | Kb | LOD | % Variance explained | Median  YPS | Median  CBS | *p* |
| --- | --- | --- | --- | --- | --- | --- | --- |
| *TPD3* | 1 | 126 | 1.21 | 2.88 | 1 | 10 | 0.0336 |
| *TEL1* | 2 | 55 | 1.66 | 3.93 | 9.24 | 0.21 | 0.0044 |
| RFT1 | 2 | 183 | 2.18 | 5.13 | 8.76 | 0.06 | 0.00154 |
| *RVB1* | 4 | 843 | 3.13 | 7.26 | 0.019 | 9.621 | 0.0001 |
| *SIR4* | 4 | 919 | 2.08 | 4.90 | 0.08 | 8.76 | 0.0020 |
| *PCL7* | 9 | 234 | 2.52 | 5.88 | 0.083 | 10.5 | 0.00067 |
| *KU80* | 13 | 479 | 1.44 | 3.41 | 10.17 | 0.51 | 0.0095 |
| *STE4* | 15 | 743 | 1.19 | 2.82 | 1.0 | 10.0 | 0.01969 |
