# Supplementary material for: Segregating YKU80 and TLC1 Alleles Underlying Natural Variation in Telomere Properties in Wild Yeast
Source: PLoS Genet. 2009 Sep 18;5(9):e1000659. doi: 10.1371/journal.pgen.1000659 (PMC2734985; doi:10.1371/journal.pgen.1000659)
Supplement: Text S1 — Structural analysis of the yKu heterodimer. (0.03 MB DOC) [file pgen.1000659.s010.doc]

**Text S1**

**Structural Analysis of the yKu heterodimer**

To begin to address the mechanism of the negative epistasis, we mapped the residues that varied between the CBS and YPS *YKU70* and *YKU80* alleles onto the structure of human Ku bound to DNA (Figure S4), reasoning that the negative epistasis should arise from regions where Yku70 and Yku80 variant residues co-localize. Not included in this analysis were 6 and 2 residues in the Yku70 N and C termini respectively, as these regions are absent in the human Ku crystal structure. Of note, deletion of the whole N terminal α/β domain in *S. cerevisae* neither affects heterodimerization or DNA end binding (A. Ribes-Zamora and A. Bertuch, unpublished observations). In addition, a total of 8 of the 37 Yku80 variant residues were not mapped because they corresponded to regions of insertions in the alignment of Yku80 with human Ku80. These residues were distributed throughout the structure.

We found that 13 of the 21 amino acid variants in Yku70 and 15 of the 37 amino acid variants of Yku80 mapped to the N-terminal α/β domains (Figure S4). These domains, on opposing faces of the heterodimer, do not contact each other. They make only minor contributions to the dimer interface and DNA binding channel, and, therefore, these variations are unlikely to affect heterodimerization or DNA binding. More notably 10 variant Yku80 and 2 variant Yku70 residues localized to the DNA ring (Figure S4). The combination of these residues could impact on Ku’s ability to properly bind telomeric ends. In addition, a cluster of variant residues in the Ku70 β barrel and Ku80 C-terminal arm were also found suggesting that these variations might impact on heterodimerization.
